# Parental Intention to Support the Use of Computerized Cognitive Training for Children With Genetic Neurodevelopmental Disorders

**DOI:** 10.3389/fpubh.2018.00309

**Published:** 2018-10-24

**Authors:** Nigel Robb, James Northridge, Yurgos Politis, Bo Zhang

**Affiliations:** ^1^Center for Global Communication Strategies, University of Tokyo, Tokyo, Japan; ^2^Centre for the Integration of Research, Teaching and Learning, University College Cork, Cork, Ireland; ^3^School of Mechanical and Materials Engineering, University College Dublin, Dublin, Ireland; ^4^Faculty of Education, East China Normal University, Shanghai, China

**Keywords:** intellectual disability, cognitive training, developmental disabilities, theory of planned behavior, assistive technology

## Abstract

Children with genetic neurodevelopmental disorders (NDDs) such as Down syndrome, Prader-Willi syndrome, and Fragile X syndrome may show a range of cognitive impairments, including impairments in executive functions (EF). EF are related to general intelligence, academic achievement, and literacy and mathematical skills. EF deficits are linked to a variety of clinically and socially important behaviors. Therefore, methods for improving EF in children with NDDs could be beneficial. One method for improving EF is through cognitive training. Research on commercial brain training programmes and video games suggests that EF can be improved through training, both in healthy adults and in children with NDDs. Computerized cognitive training (CCT) therefore represents a potentially viable intervention for children with NDDs. For training to be effective, it is important that an appropriate regimen is followed. Since children are likely to engage with training at home, the intentions of their parents to support them are therefore important. However, no research has investigated the attitudes of parents of children with NDDs to CCT. To address this, we developed a questionnaire based on the theory of planned behavior, which states that a person's intention to engage in a behavior is predicted by (1) their attitude toward the behavior, (2) their perception of subjective norms regarding the behavior (i.e., perceived social pressure), and (3) their perceived control over the behavior. The questionnaire was completed by parents of children with NDDs; 58 unique responses were retained for analyses. Parents reported low levels of knowledge of CCTs, and low levels of experience with CCTs (both their own experience and their child's experience). However, our results also show that parents of children with NDDs have positive beliefs about the potential of CCT to benefit their children and intend to support the use of CCT by their children. Linear modeling showed that, of the three constructs of the theory of planned behavior, only attitudes significantly predicted intention. Finally, parents' beliefs about the benefits of CCT correlated positively with positive attitudes toward such training. We also found limited evidence that parents of boys have more positive attitudes regarding CCT than parents of girls.

## Introduction

During early life, the typical child's brain develops rapidly. In response to both genes and the environment, neural connections develop that will play a role in every aspect of life: sensing, reasoning, language, motor skills, personality, and memory. Of particular importance is the development of executive functions, such as working memory, inhibition, and task switching (1, 2). Executive functions are high-level cognitive processes which control how and when lower-level cognitive processes operate, such as when we are multitasking or regulating our behavior (e.g., forcing ourselves to eat healthily when we would rather eat cake). Executive functions are essential for success in almost every aspect of our lives; they are related to school readiness (3), the development of academic skills (4), social-emotional competence (5), and psychological well-being (6). Deficits in executive function are associated with clinically significant behaviors such as externalizing behavior (7) and temper outbursts (8). In addition, executive functions are highly adversely affected by stress, sleep deprivation, and poor physical health (9).

Given their importance for success in so many aspects of life, the prospect of improving executive functions through training has recently received a great deal of attention. While far from conclusive, evidence from some randomized controlled trials shows the potential of a variety of training programs (9). Note that executive function training need not necessarily be provided via computer software. For example, Lakes and Hoyt (10) found that 3 months of Tae Kwon Do training improved self-regulation skills in children. However, a large proportion of research in this area has focused on a software-based approach. In such computerized cognitive training (CCT), trainees use a computer or touchscreen device to complete tasks which are designed to engage the target cognitive skills. For example, trainees may be required to categorize objects according to their color, then be asked to switch to categorizing the same objects in terms of their shape (11). This would—it is claimed—improve the trainees' ability to switch between different cognitive tasks, which is an example of an executive function ability (1). There may be several reasons why most research on cognitive training programmes has focused on a software-based approach. For example, the ubiquity of smartphones and other mobile devices, which makes computerized cognitive training more accessible and affordable by being distributed via apps or mobile games (12). In addition, companies like Cogmed^1^ and Lumos Labs^2^ are already delivering CCT to large numbers of paying customers (13), despite the fact that there is still no scientific consensus on their effectiveness (14, 15). This means that understanding if and how CCT is effective is research which can benefit a large number of consumers.

Children with neurodevelopmental disorders (NDDs) stand to benefit greatly from the development of effective CCT. There is evidence that children whose executive functioning is poorest show greatest improvement after training (9), and many NDDs are associated with deficits in executive functioning (16). In addition, we know that children with NDDs are at least as capable of, and interested in, using mobile apps and games as typically developing children (17, 18). Finally, research on specific neurodevelopmental disorders has established clear links between atypical neural development and real-world problems faced by children with NDDs and their families, *via deficits in executive function*. For example, children with the NDD Prader-Willi syndrome often display challenging behaviors (such as temper outbursts and repetitive questioning) following changes to their plans or routines (19). Empirical research has established that this phenomenon is partly caused by an impaired ability to switch between tasks (i.e., impaired executive functioning), which correlates with atypical neural activation during task switching in children with the syndrome (20, 21). More generally, this research on Prader-Willi syndrome demonstrates an approach to modeling the relationship between specific cognitive deficits and behavioral profiles in NDDs in a way which can aid the development of targeted interventions (8, 22), including the development of targeted CCT programs (11). There is therefore compelling evidence that, if CCT of executive functions can be effective, this would be especially beneficial to children with NDDs (16).

However, there are still many issues to be addressed to understand if and how CCT can be effective, both for the general population and for children with NDDs. Here we focus on a specific issue which has so far received limited attention. It is obvious that, for CCT to be effective, participants must actually take part in the training; a CCT app cannot benefit the child who does not download and use it. Yet, for a variety of reasons, ensuring engagement with CCT may not be straightforward. Firstly, during some trials of CCT programmes, researchers note that it can be difficult for trainees to adhere to the training over the required period, and therefore they feel they need to incorporate a parent or other facilitator into the process to ensure adherence (23, 24). In fact, one widely-used commercial program assigns users a coach who monitors their engagement with the software and assists with ensuring motivation and training adherence throughout.^3^ Secondly, beliefs that apps and games may have negative implications for children's health, development, or safety could impact on parents' willingness to allow their children to use CCTs (25, 26). In both these cases, we can see that parents will have a role to play in supporting the use of CCT by their children with NDDs. As such, it is important to understand the factors which may influence if and how parents of children with NDDs would support the use of CCT by their children.

The theory of planned behavior (27) may provide a systematic way to understand these behavioral intentions. The theory states that an individual's intention to perform (or refrain from performing) a behavior is predicted by (1) their attitudes toward the behavior, (2) their perception of social norms regarding the behavior, and (3) their perceived control over the behavior (27, 28). The theory provides a formal framework for the intuitive idea that the extent to which we engage in an activity (such as playing video games) is linked to our attitudes (e.g., playing video games is good for you), our perceptions of what others think (e.g., other people think it's okay to play video games), and our perceived ability to control the activity (e.g., I think I can stop myself from playing games too often). The theory has been used in a broad range of contexts and is widely regarded to provide a robust model of how an individual's behavioral intentions are determined (29). Importantly, the theory of planned behavior has also been used to successfully examine the behavioral intentions of parents and carers of people with cognitive disabilities (18, 30). Finke et al. (18) investigated the intention of parents of children with the NDD autism spectrum disorder to support video game play by their children. The authors found that parents' attitudes toward video games most strongly predicted their intention to support game play. They also showed that these attitudes were significantly positively correlated with positive behavioral beliefs regarding the effects of gameplay on their children's development, including cognitive development. While these results may have some applicability to the issue of parental intention to support CCT by children with NDD, some important caveats should be noted. Firstly, the focus of this previous study is solely on video games. While it is highly plausible that playing such games may have positive effects on cognitive processes (31), our focus is on the beliefs and intentions of parents toward software that is explicitly marketed and distributed with the intention of providing cognitive training. Most popular video games are not marketed and distributed in this way. Secondly, and most importantly, this previous study is focused only on parents of children with autism, and the cognitive profile of autism is complex: there is evidence that many individuals with autism have average intellectual ability (32), and that executive function deficits, although they may occur, are not universal in autism (32, 33). In addition, by focusing on autism, one would expect (and this was the case) more responses from parents of boys than girls, as autism is more prevalent in males (32).

In the present study we measured the attitudes, knowledge, and experience of parents of children with NDDs associated with intellectual disability regarding CCT. Our primary aim was to investigate if the constructs of the theory of planned behavior predicted parental intention to support the use of CCT. In addition, we aimed to investigate potential correlations between attitudes, knowledge, or experience regarding CCT, and correlations between the constructs of the theory of planned behavior. Our final aim was to investigate if there are differences in these attitudes, knowledge, and experience between parents of children with different identified gender or a different diagnosis of NDD.

## Materials and methods

### Questionnaire

An online questionnaire was developed for this study (see Supplemental Material for the full questionnaire). Introductory pages explained the research to respondents, making clear that their responses would be recorded anonymously and that they were not obliged to take part. This introductory section also included a brief explanation of CCT, including images showing examples of a variety of CCT programs. The images used were all obtained from the websites of CCTs; only images designated as freely-available for press use were used in the questionnaire. As in previous similar research investigating parental attitudes to technology use (18), the questionnaire incorporated items designed to determine respondents' *behavioral intention* to support CCT, *attitudes* toward CCT, their perceptions of *behavioral control* over the use of CCT and their perceptions of *social norms* regarding CCT (i.e., the constructs of the theory of planned behavior). The questionnaire also incorporated items related to behavioral beliefs about how CCT may affect their child's cognitive skills (e.g., “I believe that cognitive training programmes would help my child develop his/her problem-solving skills”), items relating to parents' knowledge of CCT, their level of experience (and their child's level of experience) with CCT, and demographic questions, including the specific NDD the respondent's child had been diagnosed with, the age of diagnosis, and their child's age and gender. Apart from demographic questions, all items were 7-point Likert-style ratings (see Supplemental Material for the labels used for each item).

### Participants and recruitment

The questionnaire was completed by parents of children with NDDs. A total of 62 responses were received. We removed 3 responses which we suspected to be duplicates. We suspected they were duplicates because each response was identical to another response and submitted very close in time to the other response. We also removed 1 response as it was unclear if the child had in fact been diagnosed with a genetic syndrome associated with ID. Participants were recruited by firstly contacting organizations that support children with NDDs. Once an organization agreed to assist with recruitment, they were asked to circulate a link to the questionnaire via email, social media, and organizations' own websites. All data was collected anonymously. All recruitment and data collection procedures were approved by the Human Research Ethics Committee at University College Dublin.

### Data preparation

Data were analyzed using IBM SPSS v. 24. To determine internal consistency of the theory of planned behavior constructs, we calculated Cronbach's alpha for each construct. A value of alpha >0.70 shows an acceptable level of internal consistency (34). Cronbach's Alpha was acceptable for attitude (a = 0.933), subjective norms (a = 0.936), and perceived behavioral control (a = 0.736). Cronbach's Alpha was also acceptable for the additional behavioral beliefs construct (a = 0.963). For each individual parent's responses, we then calculated the mean score for each of the constructs and used these means in our analyses.

## Results

The final sample consisted of 58 participants (34 female; mean age 9.38 years, std. dev. 5.94 years). The NDDs represented among the sample are shown in Table 1. Means and standard deviations for the main constructs/items are shown in Table 2: overall, parents intend to support the use of CCT by their children; their behavioral beliefs about CCT are positive, as are their attitudes toward CCT; parents perceive that social norms regarding CCT are positive; they also believe that they could control how often their child uses CCT. However, parents reported that both they and their child have limited experience with CCT. Parents also reported that they have low levels of knowledge about CCT.

**Table 1:** Genetic neurodevelopmental disorders (NDDs) with which respondents' children had been diagnosed.

**NDD**	***n* (%)**
Down syndrome	16 (27.59)
Williams syndrome	14 (24.14)
22q11.2 deletion syndrome	14 (24.14)
Prader-Willi syndrome	8 (13.79)
Fragile X syndrome	3 (5.17)
Other	3 (5.17)

**Table 2:** Means and standard deviations for the main items/constructs.

**Item/construct**	**Mean**	**Standard deviation**
Parents' knowledge of CCT	2.66	1.57
Parents' experience with CCT	1.91	1.34
Children's experience with CCT	1.90	1.63
Parents' behavioral beliefs regarding CCT	5.57	1.25
Parents' perceived social norms regarding CCT	5.18	1.40
Parents' perceived behavioral control of the use of CCT	5.60	1.30
Parents' attitudes toward CCT	5.81	0.99
Parents' intention to support the use of CCT	6.17	1.05

*CCT, Computerized cognitive training*.

To determine if parents of children with different genetic syndromes offered different responses, we ran Kruskal-Wallis *H*-tests. We used 5 groups: Prader-Willi syndrome, Down syndrome, 22q11.2 deletion syndrome, Williams syndrome, and Other. We found no significant differences between these groups in any of the constructs of the theory of planned behavior, nor in terms of parents' knowledge of CCT, parents' experience of CCT, or children's experience of CCT.

Next, we ran Mann-Whitney *U*-tests to determine if there were differences in terms of child's gender. Only the attitude construct was statistically significantly different; in this case, the distributions were judged to be similar (based on visual inspection of a population pyramid), and median attitudes scores were higher in parents of males (6.33) than females (5.66), *U* = 571, *z* = 2.584, *p* = 0.01.

Automatic linear modeling in SPSS version 24 was used to determine the direct predictors of parents' intention to support the use of CCT, based on the theory of planned behavior. Automatic data preparation was disabled, meaning that all 58 cases were included in the model and no variables were transformed. The three constructs of the theory of planned behavior (attitudes, perceived social norms, and perceived control) were included in the model. The variable selection method was forward stepwise regression. The model was significant [adjusted *R*^2^ = 0.575, *F*_(2, 55)_ = 39.585, *p* < 0.001]. Only attitudes significantly predicted intention to support CCT, as shown in the model summary in Table 3.

**Table 3:** Model summary.

**Factors**	**Coefficient**	***P*-value**	**95% confidence interval**		**Importance**
			**Lower**	**Upper**	
Intercept	1.282	0.026	0.161	2.402	
Attitudes	0.718	<0.001	0.508	0.928	0.947

*Attitudes toward computerized cognitive training (CCT) predict parental intention to support the use of CCT with their children*.

Finally, we considered correlations between variables using Spearman's Rank-Order correlation. We found a strong positive correlation between behavioral beliefs and attitudes, *r*_*s*__(56)_ = 0.715, *p* < 0.001. We also considered if behavioral beliefs or attitudes correlated with any of the following three variables: parents' knowledge of CCT; parents' experience of CCT; children's experience of CCT. None of these correlations were significant.

## Discussion

Even though cognitive training receives much attention in the media, and many such apps are widely available both online and via mobile app stores, the parents in our sample reported low knowledge of CCT. Additionally, both parents and children had limited experience with CCT. One possible explanation for this finding is that CCT programmes are typically not marketed toward children with genetic NDDs. It may be that parents of children with these syndromes—i.e., children who stand to benefit greatly from CCTs—are simply not very well informed about the applicability of such software as interventions for their children. This, in turn, may be because most current research and development on CCT is not in fact focused on children with genetic NDDs such as those exhibited in our sample. For example, Lumosity, which is one of the most widely used commercial CCT programmes (12) is only intended to be used by adults^4^, while research focusing on the application of CCT as executive function training for children with disabilities primarily focuses on attention deficit hyperactivity disorder (16).

However, despite limited knowledge of, and experience with, CCT, parents do believe that such training could potentially benefit their children, across a broad range of areas, including social skills, motor skills, cognitive skills, and quality of life. In line with previous research, we found that the theory of planned behavior can be used to model how these positive beliefs influence parents' intention to support the use of CCT: as expected, we found that attitudes were the strongest predictor of behavioral intention. In fact, in our model, the attitudes construct was the only significant predictor of intention. Furthermore, these positive attitudes are strongly correlated with parents' positive beliefs about the effects of CCT on the range of skills mentioned above. As such, our study shows that parents' intention to support the use of CCT is based primarily on positive beliefs about what CCT can achieve and positive attitudes about CCT in general.

Interestingly, our research suggests the possibility that parents' beliefs about the benefits of CCT are not necessarily based on practical experience or awareness of such programs, since knowledge of and experience with CCT were low, while beliefs and attitudes regarding CCT were high. We were unable to establish significant correlations between knowledge or experience and the variables related to planned behavior. Therefore, while it is encouraging to know that the parents in our sample believe CCT to be potentially beneficial, a crucial open question remains about what these beliefs are in turn based on.

Unexpectedly, we found limited evidence that parents of boys had significantly more positive attitudes regarding CCT than parents of girls. It would be unwise to draw conclusions based on this limited finding in a small sample. However, if parental attitudes regarding CCT were different for parents of boys and girls, this would be an important issue, as research suggests that parents' (35) and teachers' (36) gender stereotypes regarding children's abilities can negatively impact differences in attitudes and abilities of children to their education.

Our study also adds to previous research by providing evidence that the theory of planned behavior may be used to model how parents of children with NDDs *other* than autism make decisions about software use. This is important because children with NDDs linked to the genetic syndromes found in our sample will undoubtedly have different cognitive profiles to children with autism.

### Limitations

It is important to be cautious when drawing conclusions from this study due to some limitations. As our questionnaire was delivered online, our results are therefore subject to the inherent limitations of this approach. Specifically, it is recognized that technical problems can affect online questionnaires (37). It is possible that such technical problems led to the possible duplicate responses which were removed, as explained in section Participants and Recruitment. Additionally, our sample size is small, and so we should be careful about generalizing our findings. However, it is worth noting that the syndromes featured in our sample are all considered to be rare [e.g., Whittington et al. (38) estimated that the population prevalence of Prader-Willi syndrome could be as low as 1:52,000], and, as far as we are aware, our study is the first to investigate the attitudes of parents of children with NDDs to CCT. As such, it provides an important starting point for future research in this area. The final limitation is more complex. As our findings show, parents had limited knowledge of CCT. As we expected this, we prefaced our questionnaire with a brief description of CCT, including pictures illustrating specific programs. While we aimed to ensure that (1) we did not encourage parents to answer the questionnaire with a specific CCT program in mind, and (2) we did not predispose parents to have positive or negative attitudes to CCT, it is nevertheless possible that parents were influenced to some extent by this description. However, given the limited knowledge parents have of CCT, it is difficult to see how we could have avoided providing some description. This limitation could be partly addressed by conducting a similar study with parents of children with NDDs who have significant experience of CCT; such parents would presumably not require a pre-questionnaire explanation, and so it may be that the issue of influencing parents' beliefs in these introductory explanations could be avoided.

### Future work

Perhaps the clearest avenue for future work arising from our study is the open question of what exactly parents' positive beliefs about CCT are based on. It is conceivable, for example, that parents may be influenced by the claims of developers of commercialized CCT about the effectiveness of their products. This would be a concern, as Lumos Labs, a major developer of CCT, has previously been fined a substantial amount by the Federal Trade Commission for deceptive advertising^5^. In this context, it would also be interesting to carry out further research on families for which knowledge of, and experience with, CCT is higher, to determine if increased awareness of CCT impacts parental attitudes, beliefs, and intentions. It is important that parents of children with NDDs can make informed decisions about any intervention they choose to use for their child. As such, increasing the availability of clear, simple information about the effectiveness (or otherwise) of CCT, and ensuring that developers of commercial CCTs do not mislead parents of children with NDDs should be recognized as an important issue to be addressed alongside the development of potentially more effective CCT programs in the future.

## Data availability statement

The raw data supporting the conclusions of this manuscript will be made available by the authors, without undue reservation, to any qualified researcher.

## Author contributions

NR led the study, conceived the study idea and design, developed the questionnaire, carried out statistical analyses, interpreted results, and prepared the final manuscript. JN contributed material for the literature review, helped with recruitment, contributed to the development of the methods section and reviewed the manuscript. YP contributed material for the literature review, processed the experimental data, carried out some of the statistical analysis, aided in interpreting the results and reviewed the manuscript. BZ contributed to questionnaire construction, ethical approval, and the literature review.

### Conflict of interest statement

The authors declare that the research was conducted in the absence of any commercial or financial relationships that could be construed as a potential conflict of interest.

## Footnotes

^1^https://www.cogmed.com/

^2^https://lumosity.com/

^3^https://www.cogmed.com/how-is-cogmed-different

^4^https://help.lumosity.com/hc/en-us/articles/202173060-Is-Lumosity-designed-for-children-

^5^https://www.ftc.gov/news-events/press-releases/2016/01/lumosity-pay-2-million-settle-ftc-deceptive-advertising-charges/
