# Supplementary material for: Parental Intention to Support the Use of Computerized Cognitive Training for Children With Genetic Neurodevelopmental Disorders
Source: Front Public Health. 2018 Oct 24;6:309. doi: 10.3389/fpubh.2018.00309 (PMC6207646; doi:10.3389/fpubh.2018.00309)
Supplement: Supplementary file 1 [file Data_Sheet_1.pdf]

Questionnaire used in: Robb N, Northridge J, Politis Y and Zhang B (2018) Parental Intention to Support the Use of Computerized Cognitive Training for Children With Genetic Neurodevelopmental Disorders. Front. Public Health 6:309. doi: 10.3389/fpubh.2018.00309

Computerised cognitive training programmes

You have been asked to complete this questionnaire as you have a child who has been diagnosed with a genetic developmental disorder.

Your responses will be recorded completely anonymously. You can change your mind and stop taking part at any point up until you press "submit" on the final page of the form. If you change your mind, simply close the browser window and your responses will not be recorded. After you have submitted the form, it will not be possible to identify and remove your responses.

In the questionnaire, we will refer to “your child”. This always means your child who has a developmental disorder. When responding to the statements, you should think about how each statement applies to your child who has a developmental disorder. If you have other children (who do not have a developmental disorder) you should not consider them. Thank you for taking part.

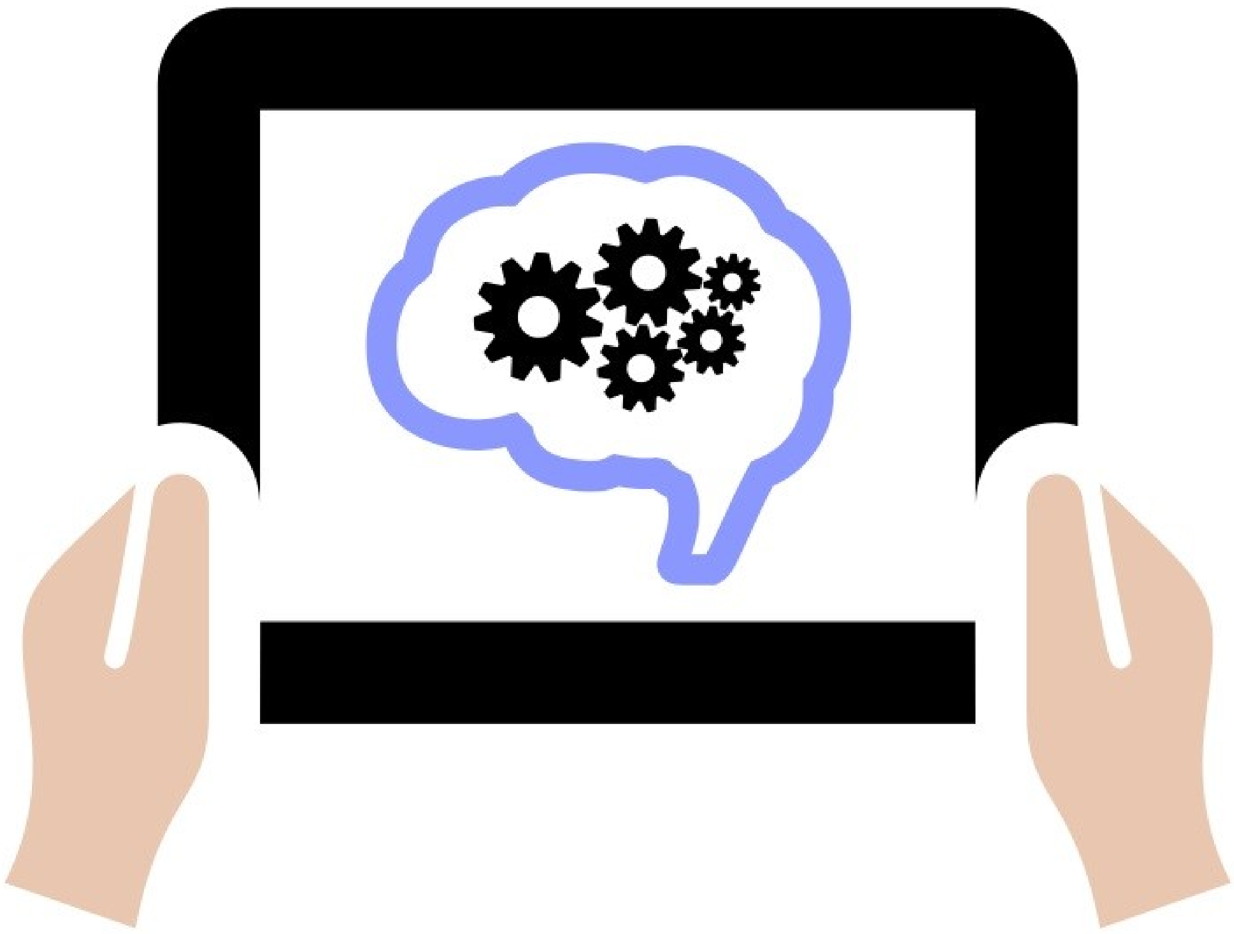

This questionnaire is about computerised cognitive training programmes. A computerised cognitive training programme is any game, app, or other piece of software that is claimed to improve cognitive

(i.e., thinking) skills. Usually, this involves completing simple games or tasks which the manufacturers claim can help develop cognitive skills. Computerised cognitive training programmes can be used on tablets, computers, games consoles, or smartphones. Some computerised cognitive training programmes you may have heard of are Lumosity and Cogmed, but there are many other such programmes available. You can see what some of these programmes look like in the images below. From now on, we will refer to these as “cognitive training programmes”.

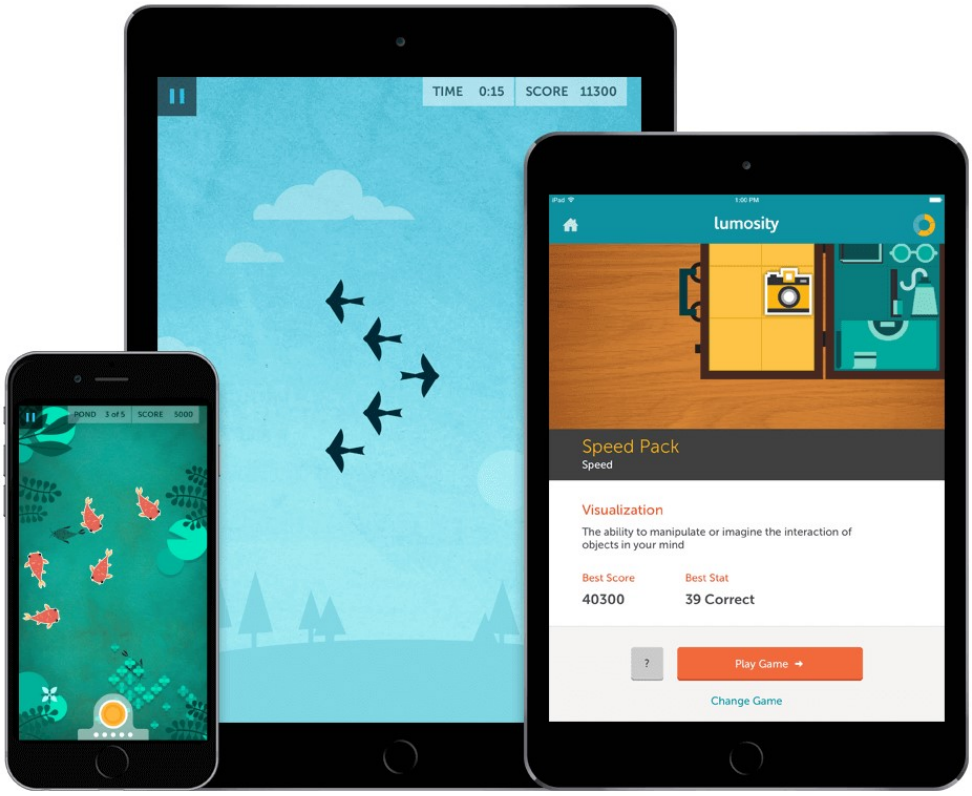

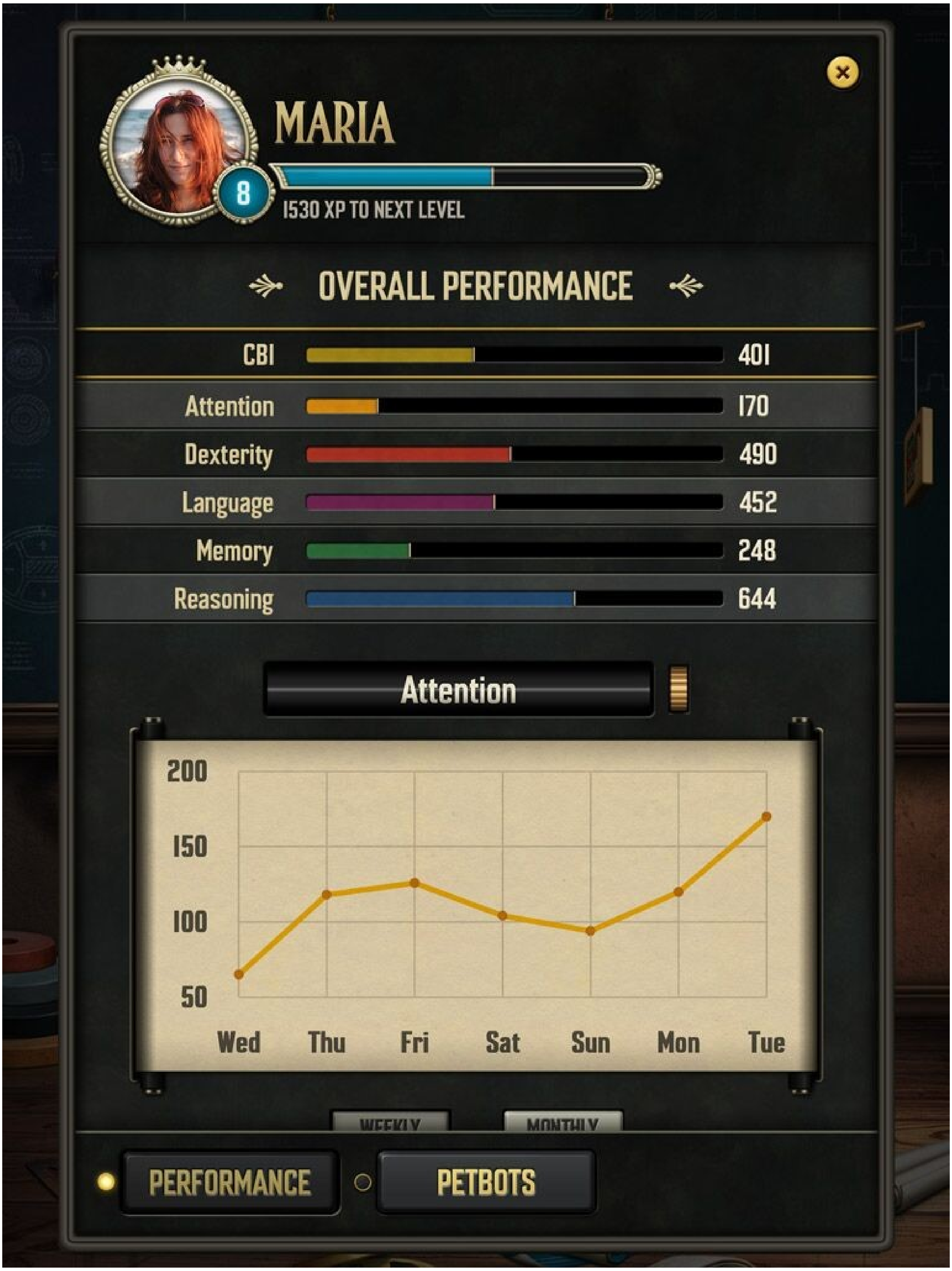

MARIA
8
1530 XP TO NEXT LEVEL
OVERALL PERFORMANCE
CBI 401
Attention 170
Dexterity 490
Language 452
Memory 248
Reasoning 644
Attention
200
150
100
50
Wed
Thu
Fri
Sat
Sun
Mon
Tue
PERFORMANCE
PETBOTS

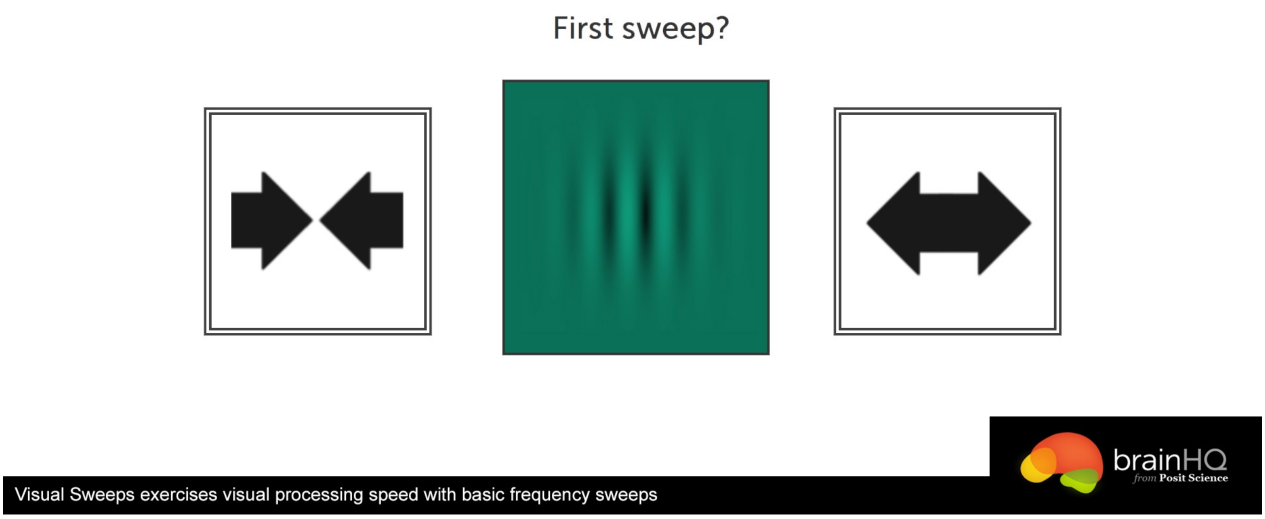

Visual Sweeps exercises visual processing speed with basic frequency sweeps

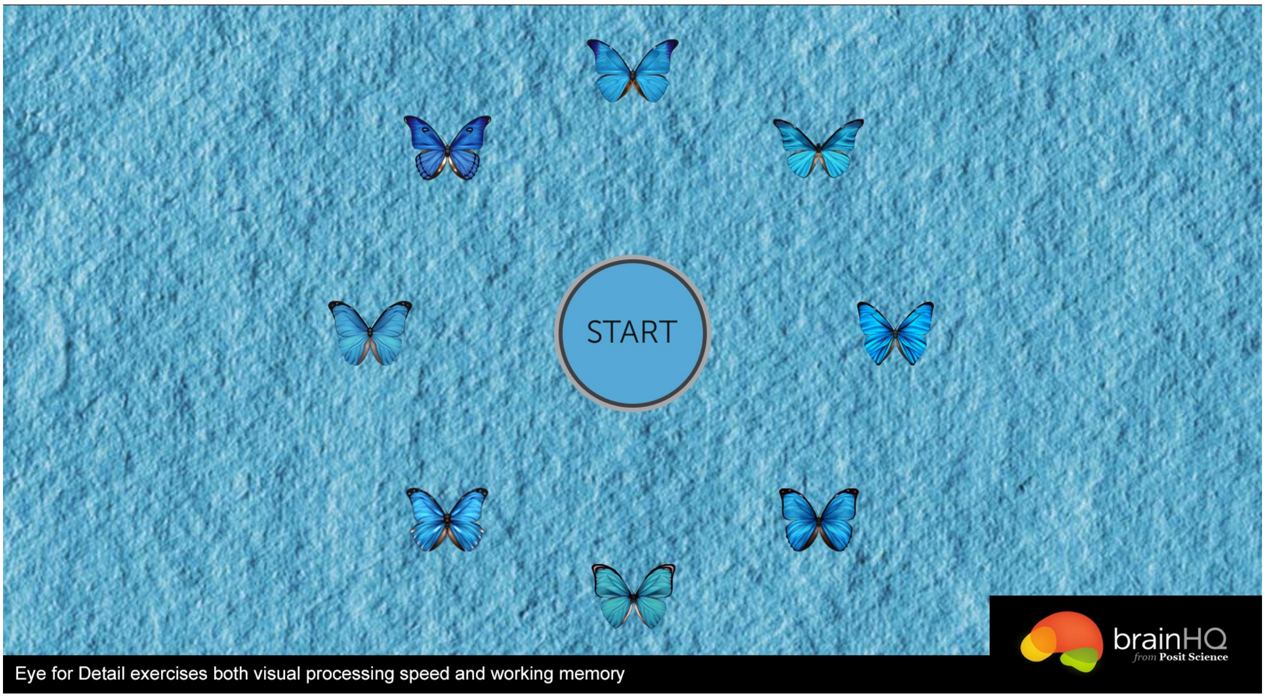

Eye for Detail exercises both visual processing speed and working memory

Featured Games

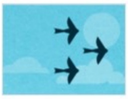

ATTENTION
Lost in Migration
Selective Attention

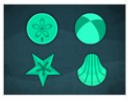

MEMORY
Tidal Treasures
Working Memory

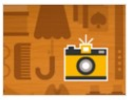

SPEED
Speed Pack
Visualization

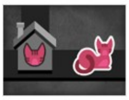

PROBLEM SOLVING
Pet Detective
Planning

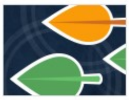

FLEXIBILITY
Ebb and Flow
Task Switching

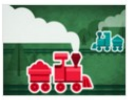

ATTENTION
Train of Thought
Divided Attention

View All Games →

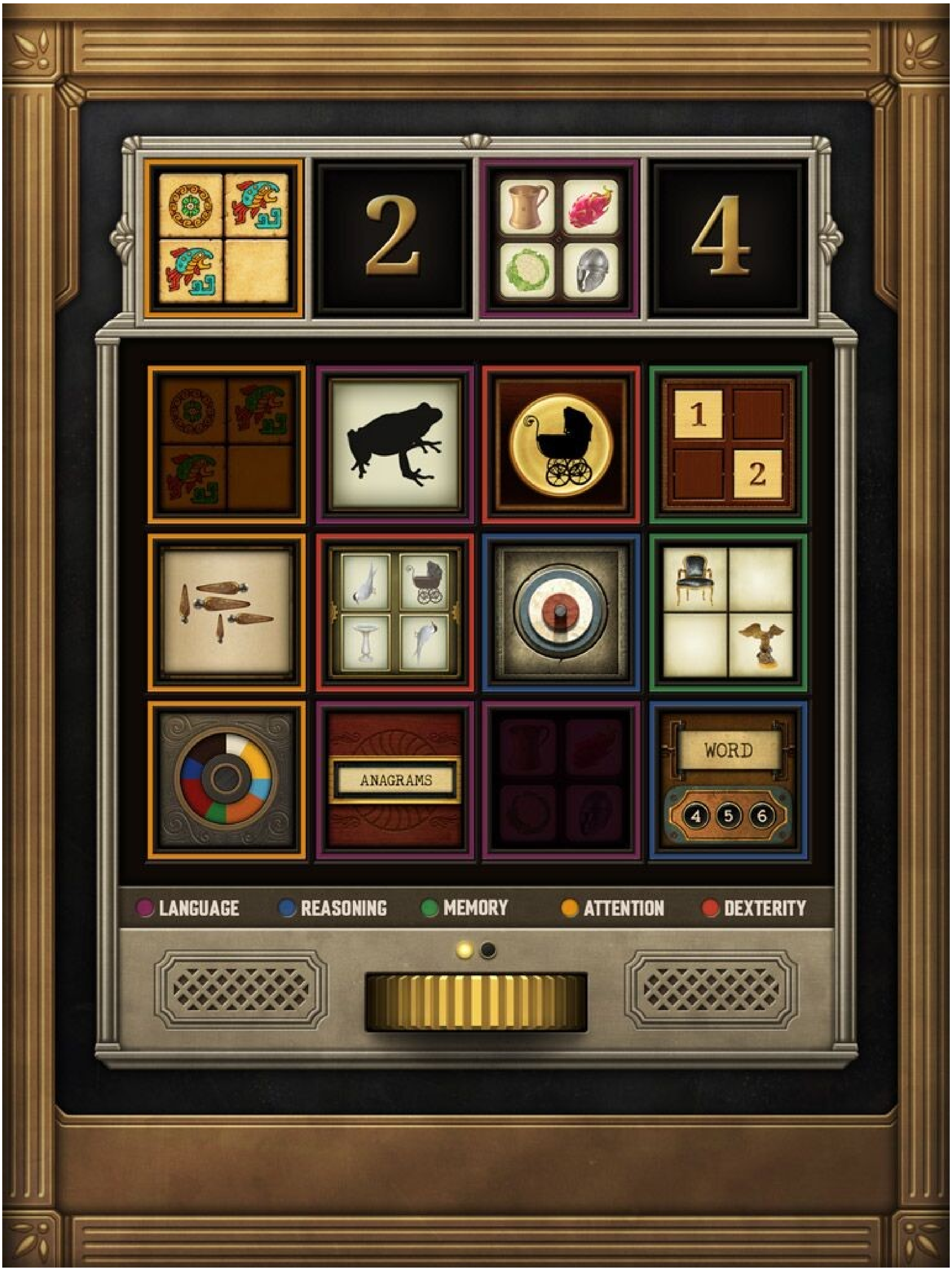
2
4
1
2
WORD
4 5 6
ANAGRAMS
LANGUAGE
REASONING
MEMORY
ATTENTION
DEXTERITY

Please answer the following questions about cognitive training programmes. For each question, select the number in the scale which you think most closely corresponds to where your answer lies between the two provided statements. For example, in the first question, if you think that the amount you know about cognitive training programmes is exactly midway between "don't know anything" and "know a lot", you would select the number 4. If you think you're closer to the "know a lot" end of the scale, select a higher number, and so on.

How much do you know about cognitive training programmes? *

	1	2	3	4	5	6	7	
I don't know anything about them	○	○	○	○	○	○	○	I know a lot about them

How much have you used cognitive training programmes? *

	1	2	3	4	5	6	7	
I've never used one	○	○	○	○	○	○	○	I use one or more regularly

How much has your child used cognitive training programmes? *

	1	2	3	4	5	6	7	
He/she has never used one	○	○	○	○	○	○	○	He/she uses one or more regularly

Please read the following statements, and indicate how much you agree or disagree with each statement by selecting a number in the scale.

I would support the use of cognitive training programmes by my child in the future. *

	1	2	3	4	5	6	7	
I strongly DISAGREE with this statement	○	○	○	○	○	○	○	I strongly AGREE with this statement

I think cognitive training programmes would contribute to the quality of life of my child. *

	1	2	3	4	5	6	7	
I strongly DISAGREE with this statement	○	○	○	○	○	○	○	I strongly AGREE with this statement

I think that cognitive training programmes would help my child develop his/her social skills. *

	1	2	3	4	5	6	7	
I strongly DISAGREE with this statement	○	○	○	○	○	○	○	I strongly AGREE with this statement

I think that cognitive training programmes would help my child develop his/her motor skills. *

	1	2	3	4	5	6	7	
I strongly DISAGREE with this statement	○	○	○	○	○	○	○	I strongly AGREE with this statement

I think that cognitive training programmes would help my child develop his/her language skills. *

	1	2	3	4	5	6	7	
I strongly DISAGREE with this statement	○	○	○	○	○	○	○	I strongly AGREE with this statement

I think that cognitive training programmes would help my child develop his/her reading/writing skills. *

	1	2	3	4	5	6	7	
I strongly DISAGREE with this statement	○	○	○	○	○	○	○	I strongly AGREE with this statement

I think that cognitive training programmes would help my child develop his/her critical thinking skills. *

	1	2	3	4	5	6	7	
I strongly DISAGREE with this statement	○	○	○	○	○	○	○	I strongly AGREE with this statement

I think that cognitive training programmes would help my child develop his/her problem-solving skills. *

	1	2	3	4	5	6	7	
I strongly DISAGREE with this statement	○	○	○	○	○	○	○	I strongly AGREE with this statement

I think that cognitive training programmes would help my child develop his/her planning skills. *

	1	2	3	4	5	6	7	
I strongly DISAGREE with this statement	○	○	○	○	○	○	○	I strongly AGREE with this statement

I think that cognitive training programmes would help my child improve his/her memory. *

	1	2	3	4	5	6	7	
I strongly DISAGREE with this statement	○	○	○	○	○	○	○	I strongly AGREE with this statement

I think that cognitive training programmes would help my child improve his/her attention. *

	1	2	3	4	5	6	7	
I strongly DISAGREE with this statement	○	○	○	○	○	○	○	I strongly AGREE with this statement

I think that cognitive training programmes would help my child improve his/her multitasking skills. *

	1	2	3	4	5	6	7	
I strongly DISAGREE with this statement	○	○	○	○	○	○	○	I strongly AGREE with this statement

Most people who are important to me would believe I should support the use of cognitive training programmes by my child. *

	1	2	3	4	5	6	7	
I strongly DISAGREE with this statement	○	○	○	○	○	○	○	I strongly AGREE with this statement

Most people who are important to me, if they had a child with a developmental disorder, would want their child to use cognitive training programmes. *

	1	2	3	4	5	6	7	
I strongly DISAGREE with this statement	○	○	○	○	○	○	○	I strongly AGREE with this statement

Most parents like me, if they had a child with a developmental disorder, would support their child to use cognitive training programmes. *

	1	2	3	4	5	6	7	
I strongly DISAGREE with this statement	○	○	○	○	○	○	○	I strongly AGREE with this statement

I am confident I could ensure that my child used cognitive training programmes for a specific number of hours each week. *

	1	2	3	4	5	6	7	
I strongly DISAGREE with this statement	○	○	○	○	○	○	○	I strongly AGREE with this statement

I am confident I could ensure that my child did not use cognitive training programmes too much. *

	1	2	3	4	5	6	7	
I strongly DISAGREE with this statement	○	○	○	○	○	○	○	I strongly AGREE with this statement

---

In this section, we would like you to read each statement, and decide where your attitude to each issue lies between the two provided words. Then select the number on the scale which represents how you feel. For example, in the first statement, if you think that children with developmental disorders using cognitive training programmes is exactly midway between "bad" and "good", you would select 4.

I think that children with developmental disorders using cognitive training programmes is... *

	1	2	3	4	5	6	7	
bad	○	○	○	○	○	○	○	good

I think that children with developmental disorders using cognitive training programmes is... *

	1	2	3	4	5	6	7	
harmful	○	○	○	○	○	○	○	beneficial

I think that children with developmental disorders using cognitive training programmes is... *

	1	2	3	4	5	6	7	
risky	○	○	○	○	○	○	○	safe

I think that children with developmental disorders using cognitive training programmes is... *

	1	2	3	4	5	6	7	
foolish	○	○	○	○	○	○	○	wise

I think that children with developmental disorders using cognitive training programmes is... *

	1	2	3	4	5	6	7	
irresponsible	○	○	○	○	○	○	○	responsible

I think that children with developmental disorders using cognitive training programmes is... *

	1	2	3	4	5	6	7	
unnecessary	○	○	○	○	○	○	○	necessary

Finally, please answer these questions about your child who has a developmental disorder

How old is your child? *

What is your child's gender? *

- Female
- Male

Which of the following syndromes has your child been diagnosed with?
*

- 22q11.2 deletion syndrome / velo-cardio-facial syndrome (VCFS) / velofacial hypoplasia / Sedlačková syndrome / DiGeorge syndrome / Shprintzen syndrome / Cayler syndrome / Conotruncal anomaly face syndrome.
- Angelman syndrome
- CHARGE syndrome
- Coffin-Lowry syndrome
- Coffin-Siris syndrome / Dwarfism-Onychodysplasia / Short Stature-Onchyodysplasia / Fifth Digit syndrome
- Cornelia de Lange syndrome
- Cri du Chat syndrome
- Down syndrome
- Fragile X syndrome
- Lesch-Nyhan syndrome (LNS) / Lesch-Nyhan disease (LND) /
- Mowat-Wilson syndrome (MWS)
- Neurofibromatosis Type 1 (NF1)
- Noonan syndrome
- Prader-Willi syndrome (PWS)
- Rett syndrome / Rett disorder / RTT
- Rubinstein-Taybi Syndrome (RTS)
- Triple-X Syndrome (47,XXX)
- Tuberous Sclerosis Complex (TSC)
- Turner syndrome
- Williams Syndrome / Williams-Beuren Syndrome
- Wolf-Hirschhorn Syndrome (WHS)
- XYY syndrome (47, XYY) / YY Syndrome /Jacob's syndrom

○ Other: ____________

How old was your child when he/she received this diagnosis? *
